# Production of Protein Hydrolysates Teff (*Eragrostis tef*) Flour with Antioxidant and Angiotensin-I-Converting Enzyme (ACE-I) Inhibitory Activity Using Pepsin and *Cynara cardunculus* L. Extract

**DOI:** 10.3390/cimb46100672

**Published:** 2024-10-11

**Authors:** Gregorio Molina-Valero, Laura Buendía-Moreno, Cindy Bande-De León, Estefanía Bueno-Gavilá, Luis Tejada

**Affiliations:** Faculty of Pharmacy and Nutrition, Universidad Católica de Murcia-UCAM, Campus de los Jerónimos, 30107 Murcia, Spain; gmolina@ucam.edu (G.M.-V.); laura.buendia.moreno@gmail.com (L.B.-M.); ebueno@ucam.edu (E.B.-G.); ltejada@ucam.edu (L.T.)

**Keywords:** bioactive peptides, hydrolyzate, teff (*Eragrostis tef*) flour, cereal, antioxidant activity, antihypertensive activity, ACE-I

## Abstract

In recent years, several studies have shown the antioxidant and antihypertensive potential of bioactive peptides. Thus, bioactive peptides are likely to be a valuable substance for the development of functional foods. There are a wide variety of sources of these peptides, including several cereals. Teff is an Ethiopian-rooted cereal with an interesting nutritional profile, mainly due to its high amount of protein. In this study, teff flour was subjected to a defatting process for optimizing the protein extraction. Such extraction was performed by precipitation from its isoelectric point, a crucial step that separates the protein from other components based on their charge. The protein obtained was subjected to enzymatic hydrolysis by pepsin and *Cynara cardunculus* L. The antihypertensive (angiotensin-I-converting enzyme ―ACE-I― inhibitory activity) and antioxidant activity (2,2-diphenyl-1-picrylhydrazyl ―DPPH― radical scavenging activity) of the peptides were determined. According to the IC_50_ values, the results obtained showed that the peptides from teff flour show promising bioactivity compared to other cereals. Furthermore, the peptides from teff flour obtained from *C. cardunculus* L. showed higher antioxidant activity (defatted teff flour ―DTF―: 0.59 ± 0.05; protein extract ―EP― : 1.04 ± 0.11) than those obtained with pepsin (DTF: 0.87 ± 0.09; EP: 1.73 ± 0.11). However, *C. cardunculus* L. hydrolyzate peptides showed lower inhibitory activity of ACE-I (DTF: 0.59 ± 0.07; EP: 0.61 ± 0.05) than the pepsin hydrolyzate (DTF: 0.15 ± 0.02; EP: 0.33 ± 0.05).

## 1. Introduction

Teff (*Eragrostis tef*), a native cereal from Ethiopia, is a unique grain that accounts for two-thirds of the protein intake in the country’s diet. Its gluten-free composition, a rare trait among cereals, has led to an increase in its global demand. This characteristic, especially significant as the diagnosis of celiac disease continues to rise, sets teff apart from other more common cereals [1,2].

Teff’s excellent macronutritional profile, with 11.4 g of protein per 100 g of product and a high proportion of complex carbohydrates, makes it a promising food for controlling blood glucose levels and reducing the risk of chronic diseases such as type II diabetes [2]. Its rich fiber content (6.8%) compared to other cereals such as rice (2.0–2.9%) or oats (2.9–3.8%) further enhances its health benefits [1,3,4]. Additionally, teff flour has an excellent lipid profile, with most of it being unsaturated (oleic and linoleic acid) [1].

Its high protein content makes teff an ideal food for obtaining bioactive peptides, defined as specific protein fragments that, as has been demonstrated in various clinical studies, have a beneficial effect on the body’s conditions and functions, thus improving our state of health [5,6]. These peptides have a size between 2–20 amino acids and have been proved to be inactive within the protein. They can be released by enzymatic hydrolysis, gastrointestinal digestion, and during the industrial processing of certain foods, presenting antihypertensive and antioxidant properties, among others [7].

Several studies have demonstrated the antioxidant and antihypertensive activity of bioactive peptides. The factors that influence the activity exerted by these peptides are diverse. Therefore, it is a complex mechanism. The structure of the peptides is one of the main characteristics that influence their bioactivity [8]; this, in turn, can change depending on the food from which they have been obtained, the enzymes used [9], and the hydrolysis conditions (time, temperature, pH, enzyme/substrate ratio, etc.) [10]. Thus, some peptides see their antioxidant activity become improved when specific amino acids are added to them [11]. Mendis et al. (2005) indicated that peptides were more likely to inhibit lipid peroxidation if hydrophobic amino acids were present in their structure, given the high interactions between the peptide and fatty acids [12].

However, the amino acid sequence is not the only feature of its structure that influences antioxidant activity. The position of amino acids within the sequence, particularly in the C- or N-terminal regions, can also be decisive. Another study found that antioxidant peptide sequences derived from different food sources often have aromatic, basic, acidic, or hydrophobic amino acids in the C- or N-terminal regions. Those tripeptides with tryptophan and tyrosine at their C-terminal end showed strong radical scavenging activity [13]. 

Molecular weight has also been reported as a feature related to the antioxidant activity of peptides. In a study using gluten hydrolysates, the antioxidant activity of peptides with a molecular weight of 500–1500 Da was higher than that of peptides above 1500 Da and below 500 Da [14].

Regarding ACE inhibitory activity, it has been reported that most of the peptides with these properties are peptides of short structure, with only two to nine amino acids. It has also been indicated that the ACE inhibitory activity was superior in those peptides whose N- or C-terminal amino acid residues had higher affinity for the active site of ACE [15]. Likewise, a study in which peptides were administered to rats showed that dipeptides with a C-terminal tyrosine residue caused a slow and prolonged decrease in systolic blood pressure compared to dipeptides with C-terminal phenylalanine. In contrast, dipeptides with C-terminal phenylalanine produced a faster reduction and shorter duration of action [16].

Various research studies have shown that bioactive peptides derived from both animal- [17] and plant-based foods [18] exhibit antioxidant and antihypertensive properties. There is a wide variety of foods from animal sources that contain bioactive peptides, ranging from cured ham [19] to marine animals. Among the latter, we find active peptides such as high Fischer ratio oligopeptides from Antarctic krill [20]; angiotensin-converting enzyme (ACE) inhibitory peptides from *Mytilus edulis* protein hydrolysate [21], emphasizing IK (Ile-Lys), YEGDP (Tyr-Glu-Gly-Asp-Pro), WF (Trp-Phe), and SWISS (Ser-Trp-Ile-Ser-Ser); antioxidant peptides from cartilage collagen of Siberian sturgeon (*Acipenser baerii*) [22]; and bioactive peptides from cardiac arterial bulbs of skipjack tuna [23], etc. Among plant source foods, we also find cereals such as wheat, oats, barley, and rice as sources of bioactive peptides. Between the sequences identified, peptides with both antihypertensive (QQPYPQ (Gln-Gln-Pro-Tyr-Pro-Gln), LQPQ (Leu-Gln-Pro-Gln), PQPQ (Pro-Gln-Pro-Gln]) and antioxidant (PYPQN (Pro-Tyr-Pro-Gln-Asn), PWQ (Pro-Trp-Gln), PHQ (Pro-His-Gln)) activity [24] can be found; these can also be obtained through enzymatic hydrolysis [25]. Bioactive peptides have been obtained from teff by fermentation [26,27]. However, there are no studies in which bioactive peptides from teff have been obtained using enzymatic hydrolysis.

To obtain bioactive peptides in cereals through enzymatic hydrolysis, enzymes from both animal [28] and plant [29] sources have been used. The use of vegetal enzymes like bromelain or papain [30,31] represents a more sustainable option since it is not necessary to slaughter animals to obtain them, as well as being more socially accepted by vegans and some religions. Among the types of enzymes that can be used to obtain peptides are plant enzymes from the thistle flower (*C. cardunculus* L.), which is a perennial herbaceous plant belonging to the Asteraceae family. This wild plant’s flowers contain two types of aspartic proteases, cynarases or cyprosins, isolated in the alkaline conditions of the stigmas, and ‘cardosins’, isolated in an acid medium [32]. Although aqueous extracts of the flower have traditionally been used for the coagulation of milk in the production of different types of cheese [33,34], these enzymes can be used in other applications, such as obtaining bioactive peptides by hydrolysis. Recently, bioactive peptides have been obtained using *C. cardunculus* L. extract from different food sources such as whey or soybean pulp, which show both antioxidant and ACE-I inhibitory activity [35,36]. Obtaining bioactive peptides from *C. cardunculus* L. extract has been poorly studied and, as stated, bioactive peptides from teff flour have never before been obtained from enzymatic hydrolysis.

On the other hand, the fact that pepsin is the protease of animal origin par excellence makes it an ideal control enzyme to evaluate the proteolytic capacity of *C. cardunculus* L. extract. Pepsin is a well-established reference enzyme in the field of food science and nutrition, particularly in studies aimed at obtaining bioactive peptides in food. It is often compared with other enzymes such as alcalase and trypsin, which are also commonly used in similar studies [37,38].

Therefore, the aim of this work is to achieve a significant milestone in the field of food science. We strive to be the first to obtain a hydrolyzate of teff flour from *C. cardunculus* L. with antioxidant and ACE-I inhibitory activity. This pioneering research could open new doors for the use of teff in functional foods and nutraceuticals.

## 2. Materials and Methods

### 2.1. Preparation of the Enzymatic Plant Extract of C. cardunculus L.

The enzymatic plant extract was obtained from the dried thistles of the species *C. cardunculus* L. (collected in Cáceres, Extremadura, Spain) using the method described by Bande-De León et al. (2023), cutting with scissors the portion of the flower that protruded from the bracts, thereby isolating styles and stigmas [39]. The plant material obtained was macerated with distilled water in a 1:10 (*w*/*v*) ratio at room temperature for 24 h. The aqueous extract was then sieved and centrifuged at 3000× *g* for 5 min. The supernatant obtained was lyophilized and stored at −20 °C. Its protein content (0.1052 ± 0.0071 mg protein/mg extract) was determined by the Bradford method [40].

### 2.2. Defatting of the Teff Flour

The teff flour was defatted following the method of Castel (2010) with slight modifications. The sample was mixed with n-hexane (Sigma-Aldrich, St. Louis, MO, USA) 95% in a 1:10 (*w*/*v*) ratio and stirred for 2 h at room temperature. It was then filtered under vacuum conditions to obtain the defatted flour, which was left to stand for 24 h at room temperature for volatilization of the residual hexane [41].

### 2.3. Teff Protein Extract Preparation

The protein extract (PE) was obtained as described by Peng et al. (2009) with some modifications followed by Tejada et al. (2022) [42,43]. The defatted teff flour (DTF) was mixed with distilled water in a 1:10 (*w*/*v*) ratio. The pH was adjusted to 8.0 with 0.5 M NaOH (ITW Reagents, Monza, Italy) to allow protein solubilization and stirred for 60 min. It was then centrifuged at 1500× *g* for 20 min. The supernatant of the tubes was adjusted to pH 4.5 with 0.1 M HCl (ITW Reagents), resulting in protein precipitation. It was then centrifuged again at 1500× *g* for 20 min. The remaining precipitate was resuspended in distilled water in a 1:5 (*w*/*v*) ratio. The pH was adjusted to 7 with NaOH and the sample was lyophilized.

### 2.4. Determination of Protein Content of Teff Flour and Protein Extract

The protein content of the PE obtained from teff flour, as well as the protein content of the DTF, was determined by the Kjeldahl method followed by Tejada et al. (2022) [43]. To carry out this analysis, a Büchi SpeedDigester Unit K-436 digestion unit (Büchi Labortechnik AG, Flawil, Switzerland) was used. To begin, 3 mL of the hydrolysate was added to the digestion tube. A protein distillation unit, Büchi MultiKjel K-365 (Büchi Labortechnik AG, Flawil, Switzerland), was then used and the sample was distilled with 70 mL of 40% NaOH (Labkem, Barcelona, Spain) and 10 mL of distilled water. The distillate was poured into an Erlenmeyer flask containing 25 mL of 4% boric acid (Carlo Erba, Sabadell, Spain) with methyl red indicator (Scharlau, Sentmenat, Spain). After distillation, titration was carried out with 0.1 N HCl.

### 2.5. Obtaining Protein Hydrolyzates

Hydrolyzates of DTF and PE were carried out by dissolving enzyme and substrate in a 1:25 (*w*/*w*) ratio using 0.03 M NaCl buffer as a solvent and adjusting the pH to the optimum for each enzyme (pH 2 for pepsin [44], pH 6.2 for *C. cardunculus* L. extract [45]), as described by Tejada et al. (2022) [43].

Tubes were placed in the thermostatic bath at the optimum temperatures for each enzyme: 37 and 50 °C for pepsin and *C. cardunculus* L. hydrolyzates, respectively. The enzyme was added and kept under hydrolysis for 16 h, according to optimal hydrolysis conditions previously established [45]. After this time, the reaction was stopped and the temperature maintained at 100 °C for 10 min. Subsequently, the pH was adjusted to 4.3 with 0.5 M NaOH and centrifuged at 1500× *g* for 20 min. The supernatant was then filtered, pH adjusted to 7 with NaOH, and frozen at −20 °C [46,47].

### 2.6. Determination of Peptide Concentration of the Hydrolyzates

For the determination of peptide concentration of hydrolyzates, the Kjeldahl method was followed [43]. To precipitate the residual protein of the hydrolyzate, 5% TCA (trichloroacetic acid; Labbox, Premià de Dalt, Spain) was added in a 1:2 ratio (*v*/*v*) and centrifuged at 1000× *g* for 20 min. 

### 2.7. Determination of Antioxidant Activity

The antioxidant activity of hydrolyzates obtained was determined by the 2,2-diphenyl-1-picrylhydrazyl (DPPH; Sigma-Aldrich, St. Louis, MO, USA) radical scavenging activity method, following the procedure described by Muñoz-Rosique et al. (2023) [19].

Dilutions were prepared at different concentrations of each of the hydrolyzates. Then, 500 μL of each dilution obtained was mixed with 500 μL of pure ethanol and 125 μL of DPPH and ethanol (Sigma-Aldrich, St. Louis, MO, USA) solution 0.02% (*w*/*v*), which was prepared at the time of the assay. This mixture was kept for 1 h in the dark at room temperature. After that time, it was centrifuged for 3 min at 10,000× *g*. Finally, it was measured in a UV-1800 (Shimadzu, Kyoto, Japan) spectrophotometer at a wavelength of 517 nm.

The percentage of DPPH radical scavenging activity (RSA) was determined by the following formula:

(1) DPPH RSA%=(Abs control−Abs sample)/Abs control×100

where:

- Abs control is the absorbance of the DPPH radical in the presence of water instead of hydrolyzate.
- Abs sample is the absorbance of the DPPH radical with the hydrolyzate.

The Trolox (Sigma-Aldrich, St. Louis, MO, USA) equivalent antioxidant capacities (TEAC; μM Trolox/mg peptides) of the hydrolyzates were also calculated. For this purpose, the percentage of antioxidant activity against the DPPH radical was represented graphically based on the concentration of Trolox (2–70 μM), using the equation of the line obtained for the interconversion, being y = 1.3664x + 0.3593, with an R^2^ = 0.999.

### 2.8. Determination of ACE-I Inhibitory Activity

In order to determine the ACE-I inhibitory activity of the hydrolyzates, the procedure described by Sentandreu and Toldrá was followed with slight modifications [48]. This procedure is based on the fluorescence emitted by hippuric acid, a product of the hydrolysis of the substrate hippuryl-histidyl-leucine (HHL) carried out by ACE-I. 

For this purpose, 50 µL of each dilution and 50 µL of 7.5 µg/mL ACE (Sigma-Aldrich, St. Louis, MO, USA) solution in 150 mM Tris (Sigma-Aldrich, St. Louis, MO, USA) buffer pH 8.3 were added to a 96-well plate. Before reading, 200 µL of 0.45 mM substrate solution (o-Aminobenzoylglycyl-p-nitro-L-phenylalanyl-L-proline; Sigma-Aldrich) in 150 mM containing NaCl (Labkem, Barcelona, Spain) 1.125 M Tris buffer pH 8.3 was added to each well [48]. The equipment used was the plate reader SpectraMax^®^ iD5 (Molecular Devices, LLC., San Jose, CA, USA) and fluorescence was measured at the time of substrate addition and 1 h later, with wavelength conditions of 375 nm (excitation) and 430 nm (emission). The ACE inhibitory activity was determined applying the following equation:

(2) $$\mathrm{ACE}\;\mathrm{inhibitory}\;\mathrm{activity}\;(\%)=(({\mathrm{FC}}_{60}-{\mathrm{FC}}_{0})-({\mathrm{FS}}_{60}-{\mathrm{FS}}_{0}))/({\mathrm{FC}}_{60}-{\mathrm{FC}}_{0})\times 100$$

where:

- FC_60_: The fluorescence emitted by the control after 60 min.
- FC_0_: The fluorescence emitted by the control at the beginning.
- FS_60_: The fluorescence emitted by the sample after 60 min.
- FS_0_: The fluorescence emitted by the sample at the beginning.

### 2.9. Statistical Analysis

All experiments were conducted in triplicate, and the results were expressed with the mean and standard error. The statistical analysis of different parameters was computed using the SPSS version 21.0 software package (IBM Corporation, Armonk, NY, USA). In order to assess differences between the samples, a one-way analysis of variance (ANOVA) was applied to protein content of the substrates. In order to assess differences between the hydrolyzates, a two-way ANOVA was performed to study the influence of enzymes and substrates. Tukey’s HSD test (*p* < 0.05) was performed to determine significant differences between the samples. Differences were considered statistically significant when *p*-values were below 0.05. 

## 3. Results and Discussion

It should be pointed out that the results of this study have been performed using in vitro assays. The conclusions obtained from them should be supported by studies carried out with cell cultures. Although it is true that the studies analyzing the antioxidant activity of peptides from cereals are not particularly large in number, there are some examples. Karami et al. (2019) [49] studied the antioxidant and ACE inhibitory activity, among others, of peptides from wheat germ. Esfandi et al. (2019) [50] studied the antioxidant activity of peptides from oats in liver cells subjected to oxidative stress. Mineo et al. (2021) [51] did the same with peptides from rice, applied in epithelial cells. Moreover, other bioactivities attributable to the peptides have been explored, such as anti-inflammatory or inhibitory activity of dipeptidyl peptidase IV (DPP-IV) [52,53].

### 3.1. Protein Characterization of Defatted Teff Flour and Its Protein Extract

The determination of the protein content of DTF and PE showed significant differences (*p* = 0.000002), supporting the efficacy of the extraction. The protein content of PE was 65.59% ± 1.23%. The protein content determined in the DTF was 13.43% ± 0.28%.

Regarding the protein content of the extract, it can be observed that the results are pretty similar to other studies. Some investigations showed a protein content of its extract around 69.7% in amaranth flour [42] and others of 64% [54] and 85% [55] in wheat flour. Therefore, it can be verified that the protein content obtained in the teff flour extract resembles most of the mentioned studies.

### 3.2. Peptide Concentration of Teff Flour and Its Protein Extract Hydrolyzates

Table 1 shows the results obtained in terms of peptide concentration after protein hydrolysis of DTF and PE by *C. cardunculus* L. and pepsin.

It can be observed that the hydrolysis capacity of *C. cardunculus* L. extract was similar in both samples. The hydrolysis capacity of pepsin was similar to C. *cardunculus* L. in DTF, but significantly increased in PE. This significantly higher concentration is probably due to combining a pure enzyme with a substrate free of components that may cause interactions. That conclusion has been reached in other studies [43].

### 3.3. Antioxidant Activity

When comparing the data expressed in Table 2, it was observed that the TEAC value of DTF hydrolyzates is higher than the TEAC value of PE. Since the crude meal has not undergone any purification process, these results may be justified by the probable presence of antioxidant compounds besides peptides in the DTF hydrolyzate, such as phenolic compounds, discarded during the PE obtaining process [56,57].

Furthermore, the peptide hydrolyzate concentration at which DPPH is reduced by 50% (IC_50_) was determined, as can be seen in Table 3. There are variations between the IC_50_ values of DTF hydrolyzate and PE hydrolyzate. In the same way, differences are observed in the values between hydrolyzates obtained from pepsin and *C. cardunculus* L. extract.

It was observed that the IC_50_ value for hydrolyzates obtained with *C. cardunculus* L. extract are lower than hydrolyzates obtained with pepsin. This result is probably due to the fact that the different specificity of the proteases causes the peptides generated by the plant enzyme to have higher antioxidant activity than those obtained by pepsin. Other studies have supported this finding [43].

Other studies carried out on different cereals show different IC_50_ values. Much higher IC_50_ values were obtained from rice flour. According to the study, multiple enzymes were used, alcalase, neutralase, trypsin, and flavourzyme, for which the peptide concentrations showing the IC_50_ were 14.04, 13.53, 10.83, and 9.90 mg/mL, respectively [58]. In the case of wheat, the concentration at which the peptides obtained reached the IC_50_ was 1.3 mg/mL [59]. This would imply that peptides obtained from teff flour exhibit high antioxidant activity, as they have lower IC_50_ values than all these cereals. On the other hand, in other study, peptides were obtained from barley flour, showing a DPPH radical inhibition rate of approximately 50% (48–58%) at a peptide concentration of 0.5 mg/mL, using flavourzyme and alcalase as enzymes [60]. 

There are no studies showing the obtaining of bioactive peptides from cereals using cynarases, but there are in other matrices. Bioactive peptides have been obtained using *Cynara scolymus* L. from matrices such as ovalbumin (13.03 ± 0.4 TEAC) [47], bovine casein [47] (4.35 ± 0.7), and insect meal (59.10 ± 1.4) [43]. These data show an antioxidant activity of the peptides obtained from teff flour that is superior to that of other sources containing proteins of high biological value and, therefore, are excellent for obtaining bioactive peptides using the same type of enzymes (cynarases).

### 3.4. ACE-I Inhibitory Activity

When comparing the IC_50_ values obtained (Table 4), it was observed that there are variations between DTF and PE values, as well as between hydrolyzates obtained from the gastric enzyme (pepsin) and that from the thistle plant extract (*C. cardunculus* L.).

In the case of pepsin, peptides obtained from DTF showed higher ACE-I inhibitory activity than those obtained from PE. However, the values showed by the peptides obtained with *C. cardunculus* L. in both DTF and PE are quite similar. On the other hand, it is observed that peptides obtained with *C. cardunculus* L. present a lower ACE-I inhibitory activity. This result may indicate that this plant enzyme presents a lower affinity for obtaining peptides with ACE-I inhibitory activity during the hydrolysis process compared to pepsin. In fact, some studies describe that pepsin is more likely to produce primarily hydrophobic peptides during hydrolysis [61]. These peptides have been recognized in some studies as having sequences more likely to generate ACE-I inhibitory activity [19].

Other studies carried out in different cereals show different concentrations for which the peptides obtained reach the IC_50_. From chia flour, this value was obtained at a peptide concentration of 0.128 mg/mL using pepsin [62]. With the same matrix and enzyme, IC_50_ value was reached at a concentration of 0.361 mg/mL [37].

On the other hand, Uraipong and Zhao (2016) showed much higher IC_50_ values in rice, specifically 8.8 and 9.2 mg/mL in different assays performed with alkalase [63]. Making use of the same enzyme, Wu et al. (2016) reached the IC_50_ at a concentration of 2.0 mg/mL, using sorghum as sample [64]. In the case of wheat, the concentration achieved for IC_50_ was 0.38 mg/mL [65]. Therefore, peptides obtained from teff flour would show high ACE-I inhibitory activity, as we can find examples of cereals from which peptides with higher activity have been obtained, such as chia or wheat.

However, cynarases have shown the ability to obtain peptides with ACE-I inhibitory activity in other matrices that presented remarkably low IC_50_ values, even lower than those of teff, among which we find casein (0.117 mg/mL) [47], ovalbumin (0.0695 mg/mL) [47], and insect meal (0.111 mg/mL) [43].

### 3.5. Pearson’s Chi-Squared Test

To determine the correlation between the antioxidant and ACE-inhibitory activity of the hydrolyzates, Pearson’s correlation test was performed between the IC_50_ of the samples. A value of −0.289 was obtained, indicating an inverse correlation between the two bioactivities studied. However, the *p* value was 0.711, which was not significant.

## 4. Conclusions

Our research has led to a significant breakthrough in the field of bioactivity and food chemistry. We have successfully obtained hydrolyzates from defatted teff flour (DTF) and protein extract (PE) with high antioxidant and ACE-I inhibitory activity using pepsin and *C. cardunculus* L. These findings inspire further exploration and innovation in this area. DTF hydrolyzates obtained with *C. cardunculus* L. presented a peptide concentration similar to DTF hydrolyzates obtained with pepsin. However, the latter was more effective in PE hydrolyzates. 

Peptides from DTF and PE obtained with *C. cardunculus* L. showed higher antioxidant activity than those obtained with pepsin but lower ACE-I inhibitory activity. 

Peptides from teff have promising bioactivity compared to peptides from other cereals and foods, showing a truly elevated antioxidant activity and intermediate values of ACE-I inhibitory activity.

Given the promising bioactivity of the sample presented in this work, further research in cells could provide valuable corroboration. This potential for future research instills hope for further advancements in our understanding of bioactive peptides. 

## Figures and Tables

**Table 1:** Peptides concentration in hydrolyzates (mg/mL).

	Defatted Teff Flour	Protein Extract	*p*-Value
*C. cardunculus* L.	1.41 ± 0.05 ^a^	1.64 ± 0.05 ^a^	0.000065
Pepsin	1.56 ± 0.07 ^a^	2.68 ± 0.06 ^b^	

Values are mean ± SE (*n* = 3). Peptide concentration values with different letters (^a^, ^b^) were statistically different.

**Table 2:** Antioxidant activity of defatted teff flour (DTF) hydrolyzate and protein extract (PE) hydrolyzate, expressed in TEAC (Eq µM Trolox/mg).

	Defatted Teff Flour	Protein Extract	*p*-Value
*C. cardunculus* L.	83.34 ± 4.05 ^b^	60.85 ± 3.53 ^c^	0.530002
Pepsin	69.73 ± 4.22 ^bc^	42.40 ± 2.75 ^a^	

Values are mean ± SE (*n* = 3). TEAC values with different letters (^a^, ^b^, ^c^) were statistically different.

**Table 3:** Antioxidant activity of defatted teff flour (DTF) hydrolyzate and protein extract (PE) hydrolyzate, expressed in IC_50_ (mg/mL) for DPPH radical scavenging activity method.

	Defatted Teff Flour	Protein Extract	*p*-Value
*C. cardunculus* L.	0.59 ± 0.05 ^b^	1.04 ± 0.11 ^c^	0.057029
Pepsin	0.87 ± 0.09 ^bc^	1.73 ± 0.11 ^a^	

Values are mean ± SE (*n* = 3). IC_50_ values with different letters (^a^, ^b^, ^c^) were statistically different.

**Table 4:** ACE-I inhibitory activity of defatted teff flour (DTF) hydrolyzate and protein extract (PE) hydrolyzate, expressed in IC_50_ (mg/mL).

	Defatted Teff Flour	Protein Extract	*p*-Value
*C. cardunculus* L.	0.59 ± 0.07 ^a^	0.61 ± 0.05 ^a^	0.153944
Pepsin	0.15 ± 0.02 ^b^	0.33 ± 0.05 ^b^	

Values are mean ± SE (*n* = 3). IC_50_ values with different letters (^a^, ^b^) were statistically different.

## Data Availability

Data is contained within the article.
